# Supplementary material for: Overexpression of Insulin Receptor Substrate 1 (IRS1) Relates to Poor Prognosis and Promotes Proliferation, Stemness, Migration, and Oxidative Stress Resistance in Cholangiocarcinoma
Source: Int J Mol Sci. 2023 Jan 26;24(3):2428. doi: 10.3390/ijms24032428 (PMC9916965; doi:10.3390/ijms24032428)
Supplement: Supplementary file 1 [file ijms-24-02428-s001.zip › Supplementary figures.pdf]

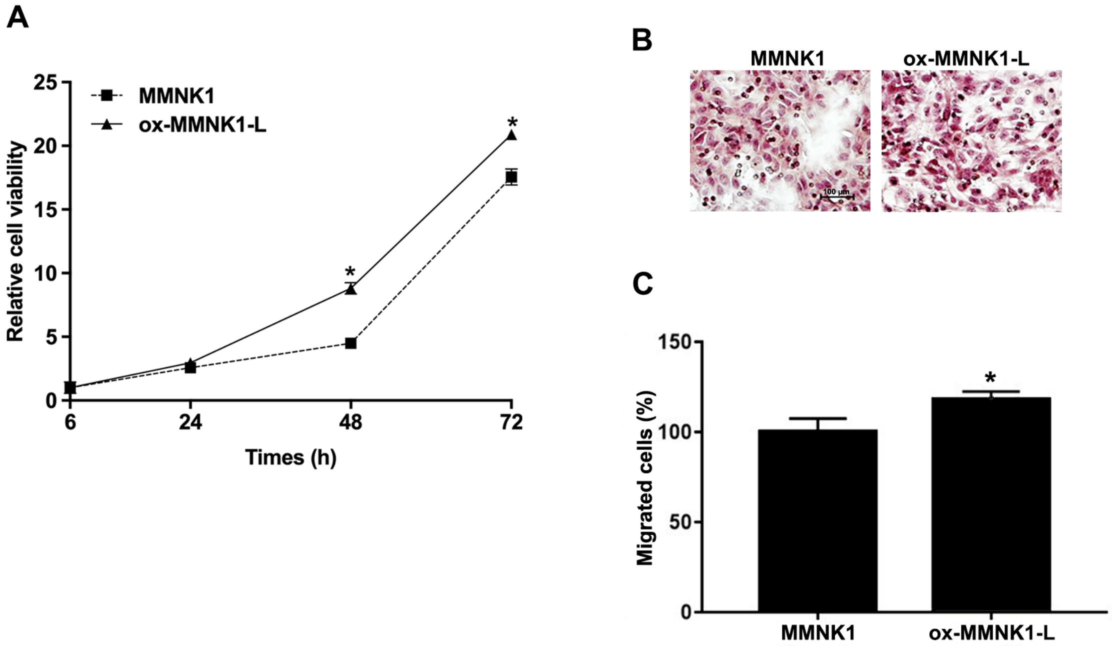

Figure S1. Cell proliferation and migration rates of MMNK1 and ox-MMNK1-L cells. (A) Relative cell viability of ox-MMNK1-L cells compared to MMNK1 cells detected by MTT assay. (B) Hematoxylin staining of migrated cells and (C) percentage of migrated cells (%) after 24 h post-migration. * p-value < 0.05 compared with MMNK1 cells.

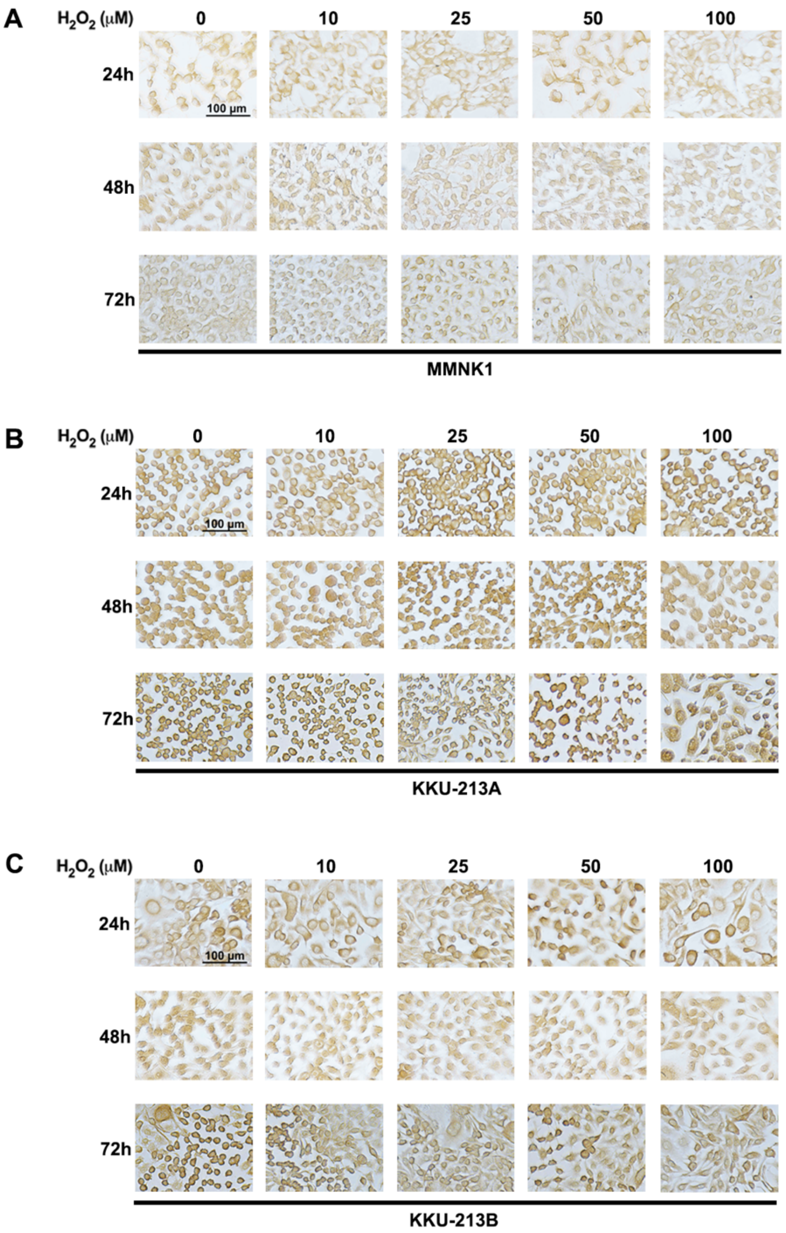

Figure S2. Effect of short-term H_2O_2 exposure on IRS1 expression in cholangiocyte and CCA cell lines. IRS1 protein expression in MMNK1(A), KKU-213A (B) and KKU-213B (C) cells after treated with H_2O_2 for 24, 48 and 72 h detected by an immunocytochemistry.

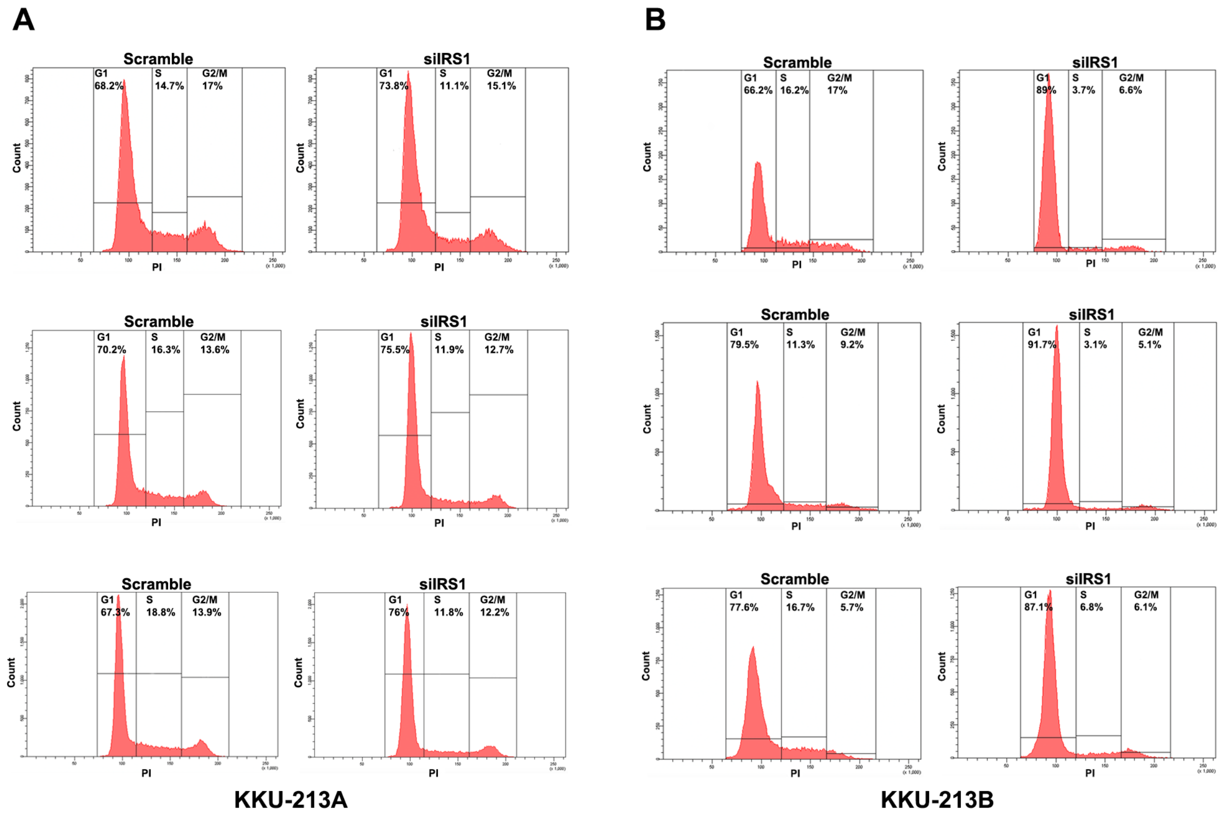

Figure S3. Effect of IRS1 knockdown on cell cycle progression of CCA cell lines. Flow cytometry results of cell distribution at different cell cycle stages after IRS1 knockdown and propidium iodide staining in (A) KKU-213A and (B) KKU-213B cells.
